# Off-Time Work-Related Smartphone Use and Bedtime Procrastination of Public Employees: A Cross-Cultural Study

**DOI:** 10.3389/fpsyg.2022.850802

**Published:** 2022-03-10

**Authors:** Wei Hu, Zeying Ye, Zhang Zhang

**Affiliations:** ^1^School of Public Administration and Policy, Renmin University of China, Beijing, China; ^2^School of Business, Sun Yat-sen University, Guangzhou, China

**Keywords:** off-time work-related smartphone use, bedtime procrastination, self-control depletion, cross-cultural study, empirical study

## Abstract

While previous studies have examined the negative effects of work-related smartphone use after hours, little is known about whether and how it influences employees’ unhealthy sleep behavior (i.e., bedtime procrastination). Drawing on the ego depletion theory, this study explored the effects of work-related smartphone use after hours on bedtime procrastination. To further uncover potential cross-cultural differences, a sample of 210 public employees from the United States and 205 public employees from China were used. Results via path analysis revealed that off-time work-related smartphone use positively influenced bedtime procrastination via the mediating role of self-control depletion. These findings were consistent between the United States and Chinese sample; however, off-time work-related smartphone use after hours increased the likelihood of self-control depletion more strongly in the United States than in China. The implications of our findings for both theory and practice were discussed.

## Introduction

Nowadays, smartphone, a type of information and communication technology, has become a ubiquitous role in our daily working lives. Many studies have shown that using smartphone for work can provide great opportunities to communicate (Kang and Jung, 2014), and enable employees to bring work tasks into the home domain thereby facilitating work flexibility (Demerouti et al., 2014). However, using smartphone for work during off-job time may not always be beneficial. Some scholars have found that off-time work-related smartphone use has dark sides for employees’ psychological health, such as work-home conflict (Derks et al., 2015, 2016; Gadeyne et al., 2018), and job burnout (Park et al., 2020). Besides, the emerging literature also found that off-time work-related smartphone use was negatively related to employees’ physical health, such as sleep quantity and sleep quality (Lanaj et al., 2014; Xie et al., 2018).

Among the research of examining the relation between off-time work-related smartphone use and employees’ physical health, it typically focused on individuals’ general sleep problems, measuring as the quantity and quality of sleep (i.e., Lanaj et al., 2014; Xie et al., 2018). Nonetheless, we still know less about the impacts of off-time work-related smartphone use on sleep behaviors, especially bedtime procrastination behavior. This is a concerning oversight given that bedtime procrastination, as individuals’ deliberately delaying going to bed or refusing to do so without external interference, is a relatively common bad behavior contributing to perceived insufficient sleep and next-day work-related outcomes (Kroese et al., 2014). In addition, smartphone has now become one of the primary channels for work and information-gathering all around the world (Kang and Jung, 2014). However, cross-cultural comparisons of the psychological effects of work-related smartphone use after hours remain scarce in the literature (Panova et al., 2020). Actually, approaching smartphone research with consideration for the influence of culture can provide insight as to why the effects of smartphone use vary in different studies. Accordingly, the current study aims to empirically examine the relationship between work-related smartphone use after hours and bedtime procrastination, and to investigate the mechanism underlying it. Besides, this study also tends to examine these relationships using two samples to uncover potential cross-cultural differences.

According to ego depletion theory (Baumeister, 2002), every volitional act (e.g., making choices, regulating emotions, and initiating or inhibiting behavior) can deplete individuals’ limited self-control capacities, leaving them with fewer self-control resources in subsequent behaviors (Hagger et al., 2010). Hence, we expect that employees’ daily off-time work-related smartphone use may influence daily bedtime procrastination through self-control depletion. On days when employees constantly handle work-related issues with their smartphone after hours, it consumes a great deal of employees’ self-control resources for task fulfillment and employees are difficult to mentally disengage from work at night. Given that bedtime procrastination is highly related to self-control ability and has therefore been conceptualized as a form of self-regulatory failure (Kroese et al., 2014), we further expect that employees with higher self-control depletion will have more difficulty going to bed at their intended bedtime.

Moreover, ego depletion theory suggests that contextual factors would influence employees’ susceptibility to resource depletion (Baumeister et al., 2007; Hagger et al., 2010). Accordingly, we further propose that cultural settings would moderate the relationship between off-time work-related smartphone use and self-control depletion. Specifically, when handling work-related issues after hours with smartphones, compared with employees in individualistic cultures, employees may have different psychological reactions in collectivist countries, where culture is heavily influenced by the principles of group interests, dedication and hierarchical obligation (Hofstede, 1980). As such, employees in collectivist countries may believe that his or her organization’s collective interest should be prioritized over his or her self-interest (e.g., hanging out with friends), being less likely to view using smartphone for work during off-job time as a threat, and thus feeling less depleted.

Overall, we seek to make several distinct contributions to the literature. First, our study complements the current literature of work-related smartphone use after hours by examining its impact on employees’ unhealthy sleep behavior (i.e., bedtime procrastination). Second, drawing on ego depletion theory, our research examines the mediating effect of self-control depletion, which addresses the relatively understudied psychological mechanism of work-related smartphone use after hours and bedtime procrastination. Third, by using two distinct samples of public employees from different cultures, we contribute to the limited body of research on the effects of work-related smartphone use after hours and self-control depletion across contexts.

## Theoretical Background and Hypotheses Development

### Off-Time Work-Related Smartphone Use

As smartphone use makes employees have permanent access to work-related information via e-mails, messengers, and phone calls, the boundary between work and home domain continually blurs (Lanaj et al., 2014). This not only poses challenges to employees in managing their work and family roles but also places increased risk on employee mental and physical health (Derks and Bakker, 2014; Derks et al., 2015, 2016; Park et al., 2020). However, whether using the smartphone for work in the evening may drive employees to exhibit more sleep-related unhealthy behaviors remains unclear. Therefore, the current study attempts to investigate the impact of work-related smartphone use after hours on bedtime procrastination and explains how and why this effect occurs.

Bedtime procrastination is a special kind of procrastination related to sleep and is defined as being unable to go to sleep as intended, excluding situations in which there is interference from external factors (Kroese et al., 2014). Previous empirical studies have shown that going to bed later than intended due to procrastination is an important risk factor for poor sleep quality (Kroese et al., 2016; Kadzikowska-Wrzosek, 2018). Bedtime procrastination often occurs when people are deeply absorbed by an activity and lost track of time (Nauts et al., 2019; You et al., 2021). A high level of work-related smartphone use after hours would induce employees to repetitively focus on their work before going to bed. This highly intensive work activity may evoke a sense of time loss, and thereby gradually increasing bedtime procrastination. Some empirical studies have provided indirect support for this inference. For example, Rozgonjuk et al. (2018) found that problematic smartphone use was consistently and positively associated with typical procrastination. Besides, a longitudinal study showed that reduced Internet use would lead to a decrease in procrastination among university students (Hinsch and Sheldon, 2013). Since bedtime procrastination is also a kind of procrastination (Kadzikowska-Wrzosek, 2018), it can be inferred that high-level work-related smartphone use after hours will cause bedtime procrastination. Accordingly, we propose:

*Hypothesis 1: Off-time work-related smartphone use is positively related to bedtime procrastination.*

### The Mediating Role of Self-Control Depletion

Self-control describes the “ability to override or change one’s inner responses, as well as to interrupt undesired behavioral tendencies (such as impulses) and refrain from acting on them” (Tangney et al., 2004, p. 274). Self-control resources are especially important because they enable individuals to behave in ways that are consistent with their own goals or organizational norms (Mead et al., 2009). As ego depletion theory suggests, whenever people engage in volitional activities, it consumes their available resources and leads to self-control depletion, which is characterized by an inability to focus or concentrate, a perceived lack of personal willpower, and a diminished capacity to exert self-control in subsequent behaviors (Baumeister, 2002; Hagger et al., 2010). Accordingly, we propose that self-control depletion would transmit the positive impact of work-related smartphone use after hours on bedtime procrastination.

Specifically, we first theorize that work-related smartphone use after hours is positively related to employees’ self-control depletion because dealing with work-related interruptions of private life requires the use of employees’ limited self-control resources. When using the smartphone for work during off-job time, employees have to control themselves to focus on tasks and reply to work-related messages or emails frequently (Derks et al., 2014). In addition, smartphone use for work at home may also trigger continuous thinking about ongoing work tasks and impending deadlines (Lanaj et al., 2014). Attempts to constrain attitudinal and behavioral reactions to these perceptions likely deplete employees’ self-control resources. Therefore, we expect that off-time work-related smartphone use increases self-control depletion.

*Hypothesis 2a: Off-time work-related smartphone use is positively related to self-control depletion.*

We further propose that employees’ self-control depletion is positively related to their bedtime procrastination. As a context-specific version of procrastination, bedtime procrastination is always regarded as a problem/failure resulting from poor self-control skills, and the failure reflects a gap between intention and behavior (Steel, 2007; Kroese et al., 2014). As for employees with depleted self-control resources, they are more vulnerable to distractions evoked by cyber-leisure activities (e.g., internet browsing, social media, gaming, etc.) (Schmeichel and Vohs, 2009; Inzlicht and Schmeichel, 2012). For instance, employees may want to relax from the work-related smartphone use and have some “me time” before going to bed. Indeed, several empirical studies have demonstrated that poor or failed self-control is an important contributor to bedtime procrastination and insufficient sleep (Exelmans and Van den Bulck, 2017; Geng et al., 2018). Therefore, we propose:

*Hypothesis 2b: Self-control depletion is positively related to bedtime procrastination.*

The above discussion suggests that off-time work-related smartphone use increases employees’ self-control depletion. Employees who suffer from self-control depletion would lack the necessary self-control resources to regulate themselves, thus exhibiting bedtime procrastination behavior. This is in line with Baumeister’s (2002) ego depletion theory, which recognizes that volitional activities will deplete individuals’ self-control resources and thus influence subsequent behaviors. Based on the above reasoning and in light of ego depletion theory, we propose the following:

*Hypothesis 2c*: *Self-control depletion mediates the impact of work-related smartphone use after hours on bedtime procrastination.*

### The Influence of Culture

Ego depletion theory posits that individuals’ perceptions of resource depletion may differ across contexts (Baumeister, 2002) and research suggests taking societal and cultural context into account whilst examining the ego-depletion effect (Savani and Job, 2017). The United States and China have traditionally been juxtaposed as individualistic and collectivist cultures (Hofstede, 1980; Earley and Gibson, 1998). Due to the vast dissimilarities between both countries, we propose that the effects of work-related smartphone use after hours on self-control depletion may operate differently across individualistic and collectivist cultures.

As Hofstede (1980) stated, individualistic cultures value the individual and emphasize autonomy and uniqueness, while collectivist cultures value the group over the individual and emphasize the need for fulfilling group goals. Particularly, people in individualistic cultures, such as the United States, prefer separating their work and non-work domains, which is in line with their independent self-construal (Lu et al., 2006). Smith et al. (1996) analyzed 43-nation data set and found that employees high in individualism had less organizational involvement. Accordingly, when employees receive work-related messages through smartphone during off-job time, those in individualistic countries may appraise is as threatening and harmful, thus feeling high self-control depleted, because dealing work issues at home with smartphone make it harder for them to spend quality time on non-work domains.

On the contrary, employees in collectivistic countries, such as China, might view work-related smartphone use as less threatening, and feel less self-control depleted. This is because employees in collectivistic countries value organizational interests over self-interests and are dedicated to work, thus they are always used to blurring the boundary of their work and non-work domains (Smith et al., 1996). In addition, compared with employees in individualistic cultures, those in collectivistic cultures are more confident that family members will help take care of some of their family responsibilities (Spector et al., 2007). Thus, work-related smartphone use after hours may be perceived as a natural extension of their daytime work. In summary, we argue that due to a higher level of individualism, the negative effects of work-related smartphone use after hours is greater for employees in the United States compared to employees in China. Consequently, the positive effect of work-related smartphone use after hours on self-control depletion is stronger in the United States than in China.

*Hypothesis 3: The positive relationship between off-time work-related smartphone use and self-control depletion is weaker in China than in the United States.*

Given that self-control depletion mediates the impact of work-related smartphone use after hours on bedtime procrastination (Hypothesis 2c) and the positive relationship between work-related smartphone use after hours and self-control depletion is weaker in China than in the United States (Hypothesis 3), it is reasonable to hypothesize that the indirect effect of work-related smartphone use after hours on bedtime procrastination is weaker in China than in the United States. Thus, we formulate an extended hypothesis.

*Hypothesis 4: The indirect effect of work-related smartphone use after hours on bedtime procrastination via self-control depletion is weaker in China than in the United States.*

## Materials and Methods

### Participants and Procedure

We collected Chinese sample via a Master of Public Administration (MPA) program in a university in China. With the help of the MPA center, we invited MPA students in one semester who were employees in public sectors to participate in our survey. We ensured that their participations were totally voluntary and their responses would be confidential. To increase the participation rate, we also promised that they would receive $10 as a compensation. The participants were required to complete an online survey that included work-related smartphone use after hours, self-control depletion, bedtime procrastination, and demographic information. Initially, 272 MPA students agreed to participate in our survey. After excluding the uncompleted cases, we obtained 205 responses as the final sample with a total response rate of 75.37%. The results of independent *t* tests showed that the missing data were completely at random. Of the 205 participants, 126 were male. Their average age was 31.73 years (SD = 7.16), and average organizational tenure was 5.16 years (SD = 5.60). Moreover, 66.83% of them had a bachelor’s degree, and the rest had a master’s degree or higher.

We collected the United States sample through Amazon Mechanical Turk (Mturk). To qualify for the study, participants had to confirm that they are employees in public sectors. The participants who unsatisfied this criterion did not proceed with the survey. All participants were compensated $10 for their response. The participants were required to self-report work-related smartphone use after hours, self-control depletion, bedtime procrastination, and demographic information. We initially received 265 responses. After excluding the uncompleted cases, the final sample had 210 responses with a total response rate of 79.25%. The results of independent *t* tests showed that the missing data were completely at random. Among the 210 participants, 122 were male. Their average age was 33.09 (SD = 9.72), and their average organizational tenure was 6.89 years (SD = 7.34). Moreover, 46.19% had an associate’s degree, 49.52% had a bachelor’s degree, and 4.29% had a master’s degree or above.

### Measures

All measures were derived from previously validated scales. We implemented the standard translation and back-translation procedures to ensure the translation quality. All items were assessed on a 5-point Likert-type scale ranging from 1 (strongly disagree) to 5 (strongly agree).

#### Off-Time Work-Related Smartphone Use

Participants rated the four-item scale developed by Derks et al. (2014). A sample item is “These days, I have to check for work-related text messages until I fall asleep.” The Cronbach’s alpha score was 0.97 in Chinese sample and was 0.96 in the United States sample.

#### Self-Control Depletion

Participants rated five items on a scale developed by Twenge et al. (2004). A sample item is “I feel like my willpower is gone.” The Cronbach’s alpha score was 0.96 in Chinese sample and was 0.96 in the United States sample.

#### Bedtime Procrastination

Participants rated the nine-item scale developed by Kroese et al. (2014). A sample item is “Often, I am still doing other things when it is time to go to bed.” The Cronbach’s alpha score was 0.97 in Chinese sample and was 0.97 in the United States sample.

#### Control Variables

We controlled for demographic variables (i.e., gender, age, tenure, and education level) to rule out their potential influences on the results.

## Results

### Common Method Bias Check

Since all measures in Chinese sample and the United States sample were self-reported, it may cause potential common method bias. Thus, we conducted Harman’s (1976) single factor test to detect common method variance. The results showed that, in Chinese sample, after unrotated exploratory factor analysis for all items, the total variance explained by factors with eigenvalues greater than one was 87.00%. Moreover, the first principal component accounted for 44.74% of the total variance, falling below the 50% cutoff point. Moreover, using the same procedure in the United States sample, the results suggested that the factors with eigenvalues greater than one explained 86.32% of the total variance and the first principal component accounted for 42.50% of the total variance, bellowing the 50% cutoff point. Taken together, these results indicated that common method bias was not a serious threat in our two samples.

### Confirmatory Factor Analysis

To assess the measurement validity of our proposed three-factor model (i.e., work-related smartphone use after hours, self-control depletion, and bedtime procrastination) in our two samples, we conducted the confirmatory factor analysis by using Mplus 7.0 software (Muthén and Muthén, 2012). In Chinese sample, the results suggested that the three-factor model indicated an adequate model fit [*χ^2^*(132) = 275.70, *χ^2^*/*df* = 2.07, CFI = 0.97, TLI = 0.96, RMSEA = 0.07, SRMR = 0.03] and had a better model fit than other alternative models (in which the three factors were combined in different ways). Moreover, in the United States sample, the results also revealed that three-factor model yielded an acceptable model fit [*χ^2^*(132) = 466.51, *χ^2^*/*df* = 3.53, CFI = 0.95, TLI = 0.94, RMSEA = 0.11, SRMR = 0.02] and had a better model fit than other alternative models (in which the three factors were combined in different ways). Taken together, these results demonstrated that our variables were adequately distinct.

### Hypothesis Testing

Table 1 presents the means, standard deviations, and correlations of the study variables. To test hypotheses, we conducted path analysis by using Mplus 7.0 software (Muthén and Muthén, 2012). As for Hypothesis 1 and 2, we divided our data into Chinese and the United States sample according to the country that participants belong to, and then conducted path analysis separately.

**TABLE 1:** Descriptive statistics and bivariate correlations.

Variable	Mean	*SD*	1	2	3	4	5	6	7
*Chinese sample* (*N = 205*)									
1. Gender	0.61	0.49							
2. Age	31.73	7.16	–0.05						
3. Tenure	5.16	5.60	0.03	0.72***					
4. Education	2.33	0.47	0.05	0.15*	0.24***				
5. Work-related smartphone use after hours	4.14	1.39	0.07	–0.02	0.05	0.07	(0.97)		
6. Self-control depletion	3.23	1.26	0.00	−0.16*	–0.10	–0.12	0.25***	(0.96)	
7. Bedtime procrastination	3.50	1.24	0.13	0.09	0.16*	0.07	0.15*	0.19**	(0.97)
*The United States sample* (*N = 210*)									
1. Gender	0.58	0.49							
2. Age	33.09	9.72	–0.06						
3. Tenure	6.89	7.34	−0.20**	0.69***					
4. Education	1.58	0.58	0.05	−0.30***	−0.20**				
5. Off-time work-related smartphone use	4.01	1.55	0.12	0.03	0.07	0.00	(0.96)		
6. Self-control depletion	3.83	1.64	0.00	–0.02	–0.00	0.01	0.39***	(0.96)	
7. Bedtime procrastination	3.37	1.44	0.04	0.02	0.01	0.05	0.28***	0.40***	(0.97)

The numbers in parentheses are Cronbach’s alpha scores. Gender: 0, female; 1, male. Education: 1, associate’s degree; 2, bachelor’s degree; 3, master’s degree or above. *p < 0.05; **p < 0.01; ***p < 0.001.

**FIGURE 1:**
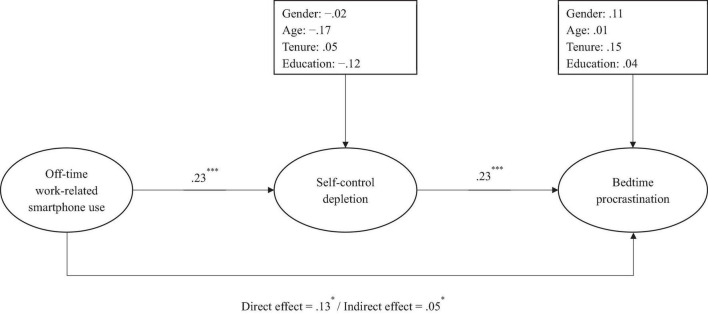
The path analysis results in Chinese sample. Standardized coefficients were reported. *p < 0.05; ^***^p < 0.001.

For the Chinese sample, the results (see Figure 1) showed that the direct effect of work-related smartphone use after hours on bedtime procrastination was positive and significant (β = 0.13, *p* < 0.05), supporting Hypothesis 1. Meanwhile, work-related smartphone use after hours has a positive and significant impact on self-control depletion (β = 0.23, *p* < 0.001), which in turn positively and significantly affects bedtime procrastination (β = 0.23, *p* < 0.001). Thus, Hypothesis 2a and 2b were supported. The bootstrapping results (Preacher and Selig, 2012) showed that the indirect effect of work-related smartphone use after hours on bedtime procrastination through self-control depletion was positive and significant [*indirect effect* = 0.05, *95% CI* = (0.00, 0.12)], thus providing support for Hypothesis 2c.

**FIGURE 2:**
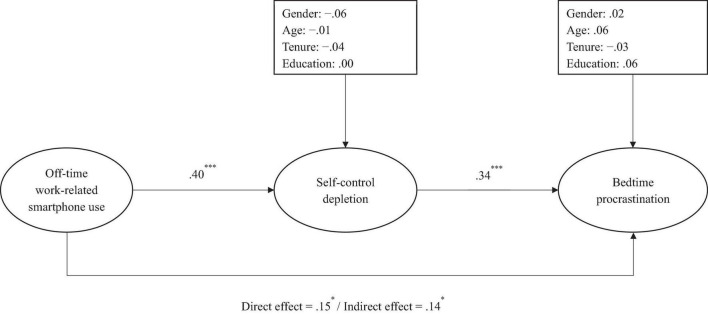
The path analysis results in the United States sample. Standardized coefficients were reported. *p < 0.05; ^***^p < 0.001.

For the United States sample, the results (see Figure 2) suggested that the direct relationship between work-related smartphone use after hours and bedtime procrastination was positive and significant (β = 0.15, *p* < 0.05), supporting Hypothesis 1. Work-related smartphone use after hours was positively associated with self-control depletion (β = 0.40, *p* < 0.001), which in turn was positively related to bedtime procrastination (β = 0.34, *p* < 0.05). Moreover, the bootstrapping results showed that the mediation effect of self-control depletion was positive and significant [*indirect effect* = 0.14, *95% CI* = (0.02, 0.27)], supporting Hypothesis 2c.

To test Hypothesis 3 and 4, we conducted multiple-group path analysis. We compared two models to investigate whether Chinese sample and the United States sample were different from each other. Specifically, in the unconstrained model, we allowed the structural paths to vary across samples. In the constrained model, we constrained all the structural paths to be same in both samples. Results of Chi-square difference test showed that the unconstrained model had a significant better model fit than the constrained model (Δ*χ^2^* = 17.62, Δ*df* = 5, *p* < 0.05). This implied that the path coefficients across samples were not equal.

Furthermore, we tested whether the difference existed between the paths in two samples. The results showed that the path from work-related smartphone use after hours to self-control depletion was weaker in Chinese sample than in the United States sample (β *_*China*_* = 0.23, β *_*the U*.S._* = 0.40, *coefficient difference* = −0.17, *p* < 0.001), thus providing support for Hypothesis 3. Moreover, the indirect effect of work-related smartphone use after hours on bedtime procrastination via self-control depletion was also weaker in Chinese sample than in the United States sample (*indirect effect _*China*_* = 0.05, *indirect effect _*the U.S.*_* = 0.14, *indirect effect difference* = −0.09, *p* < 0.05). Therefore, Hypothesis 4 was supported.

## Discussion

Drawing on ego depletion theory, this study investigated the effects of work-related smartphone use after hours on bedtime procrastination. To further explore the potential cross-cultural differences, this study collected data from public employees working in China (*N* = 205) and in the United States (*N* = 210). Results showed that work-related smartphone use after hours was positively associated with bedtime procrastination. And self-control depletion mediated this relationship. Additionally, work-related smartphone use after hours increased the likelihood of self-control depletion more strongly in the United States than in China.

### Theoretical Contributions

The current research makes three significant theoretical contributions. First, the current study adds to the nomological network of work-related smartphone use after hours. As mentioned above, prior research has typically centered on general sleep problems that off-time work-related smartphone use causes, while ignoring its detrimental effect on sleep behaviors, especially bedtime procrastination behavior (i.e., Lanaj et al., 2014; Xie et al., 2018). In the current study, we found that work-related smartphone use after hours would trigger bedtime procrastination, which responds to the call for more research relative to the negative effects of work-related smartphone use after hours on health behaviors (Kang and Jung, 2014). In doing so, we also shed light on the role of work-related factors in explaining bedtime procrastination. Although bedtime procrastination is prevalent in modern society (Kroese et al., 2014), most studies attempted to investigate this phenomenon by using student samples (e.g., Kadzikowska-Wrzosek, 2018; You et al., 2021). Only a few studies have focused on bedtime procrastination in organizational context; however, they mainly examined the antecedents of bedtime procrastination from a personal characteristic perspective (e.g., self-regulation skills, Kadzikowska-Wrzosek, 2020; boredom proneness, Teoh et al., 2021), while ignoring the potential impact of work-related factors. The current study complements the existing literature by demonstrating off-time work-related smartphone use as an important predictor of employees’ bedtime procrastination, which provides important insights into the antecedences of bedtime procrastination.

Second, anchored in ego depletion theory, our study illuminates the “black box” by empirically underscoring the importance of employees’ self-control depletion as an intervening process mechanism linking work-related smartphone use after hours and employees’ bedtime procrastination. This investigation is meaningful because it suggests that work-related smartphone use after hours would influence employees’ psychological perceptions, and thus influence employees’ sleep behaviors. In doing so, we also respond to the call for more examinations about the specific mechanisms through which work-related smartphone use after hours impacts subordinates (Derks et al., 2016). Extrapolating from this point of view, future research can examine other underlying mechanisms linking work-related smartphone use after hours and employees’ bedtime procrastination in order to understand its influence fully.

Third, the results address the gap in examining how culture influences the off-time work-related smartphone use and self-control depletion relationship by using two distinct samples of public employees from China and the United States. Specifically, the results showed that work-related smartphone use after hours does indeed increased employees’ self-control depletion in both countries. However, off-time work-related smartphone use increased self-control depletion more strongly in the United States than in China. This cross-cultural difference is not quite surprising though, considering the fact that working during off-job time is viewed differently between individualistic and collectivist cultures. But to the best of our knowledge, our study is among the first to empirically investigate the effects of work-related smartphone use after hours on bedtime procrastination across cultural settings. Hence, our consideration of cultural settings contributes important information about the contingency factors that shape the effects of work-related smartphone use after hours. Also, it enriches the understanding of how certain cultural factors influence public employees’ perceptions and responses to work-related smartphone use after hours. Future research could consider other different cultural factors influencing the effects of work-related smartphone use after hours.

### Practical Implications

Our endeavors also carry important implications for organizational management. First, our arguments indicate that using the smartphone for work in the evening increase the probability of bedtime delay. Given that bedtime procrastination may cause sleep deprivation and further negative consequences (Kadzikowska-Wrzosek, 2020; Teoh et al., 2021), organizations should establish a clear policy for off-time working communications and provide instructions on the use of electronic communication to maximize work efficiency.

Second, because self-control depletion would cause bedtime procrastination, employees should intentionally take actions to recover from depletion, thus meeting their intended bedtimes. For example, in the evening, employees can finish their chores as soon as possible, and then retain a certain amount of slack time to do what they want. In addition, employees can also ask their family members to remind them of their bedtime or just what time it is. These approaches may help them get more of the sleep they need.

Third, this study shows that the effects of work-related smartphone use after hours on self-control depletion operate differently in different cultures, influenced by the individualism/collectivism valued in their countries. In particular, we show that compared to employees in China, employees in the United States feel greater depleted due to off-time work-related smartphone use. These results suggest that managers may need to avoid assigning tasks during off-job time in the U.S. and other individualistic cultures. For Chinese and other collectivist cultures, managers should also pay attention to the psychological states of their employees, rather than blindly asking employees to continue working after hours.

### Limitations and Future Directions

Despite the contributions we make, this study has some limitations. First, the data collected in this study is cross-sectional, which raises concern that some observed relations may be biased by common method variance. And the variables measured by cross-sectional design could not obtain evidence about causality. To fully address this limitation, future research should consider utilizing various research designs (e.g., multi-time and multi-source designs, experiments or longitudinal studies), which could provide further support for the predictive validity of the current study.

Second, we measured off-time work-related smartphone use by using Derks et al.’s (2014) scale, which has been verified as a reasonable and reliable approach (Derks et al., 2015, 2016). However, to get a more concrete view of how employees actually use smartphone for work purposes during after-work hours, objective measures, such as the frequency of smartphone use or a log book, would be helpful. Therefore, we recommend future studies using these objective measures to replicate our research findings.

Third, in our theoretical argumentation, we only focused on the individualism and collectivism factor to hypothesize different effects across two countries. However, China and the United States differ along several other aspects such as size, welfare, and power distance orientation. Since we did not measure other specific cultural variables, we were unable to conclude which differences between the two samples actually contributed to the observed effects. In this case, it would be beneficial for future studies to further explore the role of cultural and other personal factors, i.e., employee conscientiousness when examining work-related smartphone use after hours.

The fourth limitation in this study concerns the generalizability of the findings. Limited by human, material, financial resources, we adopted convenience sampling method to collect data rather than random sampling. This may not be enough to represent employees in China and the United States. Besides, our data were collected only from public employees working in China and in the United States, which may also prevent the generalizability of the findings to other countries. Future research should consider additional cross-cultural models to better understand the influence of culture on the relationship between work-related smartphone use after hours, self-control depletion, and bedtime procrastination.

## Conclusion

Based on ego depletion theory, we found a positive relationship between off-time work-related smartphone use and bedtime procrastination, and a mediating role of self-control depletion. Additionally, work-related smartphone use after hours increased the likelihood of self-control depletion more strongly in the United States than in China. Our findings offer preliminary but important insights regarding how off-time work-related smartphone use is most likely to be positively or negatively related to bedtime procrastination among public employees from the United States or China. We hope that our research could allow practitioners to understand employees’ off-time work-related smartphone use in a cross-cultural perspective.

## Data Availability Statement

The raw data supporting the findings of this study will be made available by the authors to qualified researchers upon reasonable request. Requests to access the datasets should be directed to WH, cchuwei@126.com.

## Ethics Statement

The studies involving human participants were reviewed and approved by Renmin University of China. The patients/participants provided their written informed consent to participate in this study.

## Author Contributions

WH led the literature review, research design, and manuscript drafting work. ZY made contributions in data analysis. ZZ made contributions in manuscript drafting work. All authors contributed to the article and approved the submitted version.

## Conflict of Interest

The authors declare that the research was conducted in the absence of any commercial or financial relationships that could be construed as a potential conflict of interest.

## Publisher’s Note

All claims expressed in this article are solely those of the authors and do not necessarily represent those of their affiliated organizations, or those of the publisher, the editors and the reviewers. Any product that may be evaluated in this article, or claim that may be made by its manufacturer, is not guaranteed or endorsed by the publisher.
